# The Genetic Link between Parkinson's Disease and the Kynurenine Pathway Is Still Missing

**DOI:** 10.1155/2015/474135

**Published:** 2015-02-17

**Authors:** Nóra Török, Rita Török, Zoltán Szolnoki, Ferenc Somogyvári, Péter Klivényi, László Vécsei

**Affiliations:** ^1^Department of Neurology, Faculty of Medicine, Albert Szent-Györgyi Clinical Centre, University of Szeged, 6 Semmelweis Utca, Szeged 6725, Hungary; ^2^Department of Neurology and Cerebrovascular Diseases, Pándy Kálmán County Hospital, 1 Semmelweis Utca, Gyula 5700, Hungary; ^3^Department of Medical Microbiology and Immunobiology, University of Szeged, 10 Dóm tér, Szeged 6725, Hungary; ^4^MTA-SZTE Neuroscience Research Group, 6 Semmelweis Utca, Szeged 6725, Hungary

## Abstract

*Background*. There is substantial evidence that the kynurenine pathway (KP) plays a role in the normal physiology of the brain and is involved in the pathology of neurodegenerative disorders such as Huntington's disease and Parkinson's disease (PD). *Objective*. We set out to investigate the potential roles in PD of single nucleotide polymorphisms (SNPs) from one of the key enzymes of the KP, kynurenine 3-monooxygenase (KMO). *Methods*. 105 unrelated, clinically definitive PD patients and 131 healthy controls were enrolled to investigate the possible effects of the different alleles of KMO. Fluorescently labeled TaqMan probes were used for allele discrimination. *Results*. None of the four investigated SNPs proved to be associated with PD or influenced the age at onset of the disease. *Conclusions*. The genetic link between the KP and PD is still missing. The investigated SNPs presumably do not appear to influence the function of KMO and probably do not contain binding sites for regulatory proteins of relevance in PD. This is the first study to assess the genetic background behind the biochemical alterations of the kynurenine pathway in PD, directing the attention to this previously unexamined field.

## 1. Introduction

Parkinson's disease (PD) is a chronic progressive neurodegenerative disease mostly among the elderly with characteristic pathological hallmarks; selective degeneration of the dopaminergic neurones in the substantia nigra pars compacta; and the presence of Lewy bodies.

The main mechanisms behind the aetiology and pathology of PD are oxidative stress, mitochondrial disturbances, protein aggregation, excitotoxicity, immunological mechanisms, and a genetic predisposition [1–7]. Recent studies also indicate the role of the altered tryptophan (Trp) metabolism in PD [8–10].

**Figure 1:**
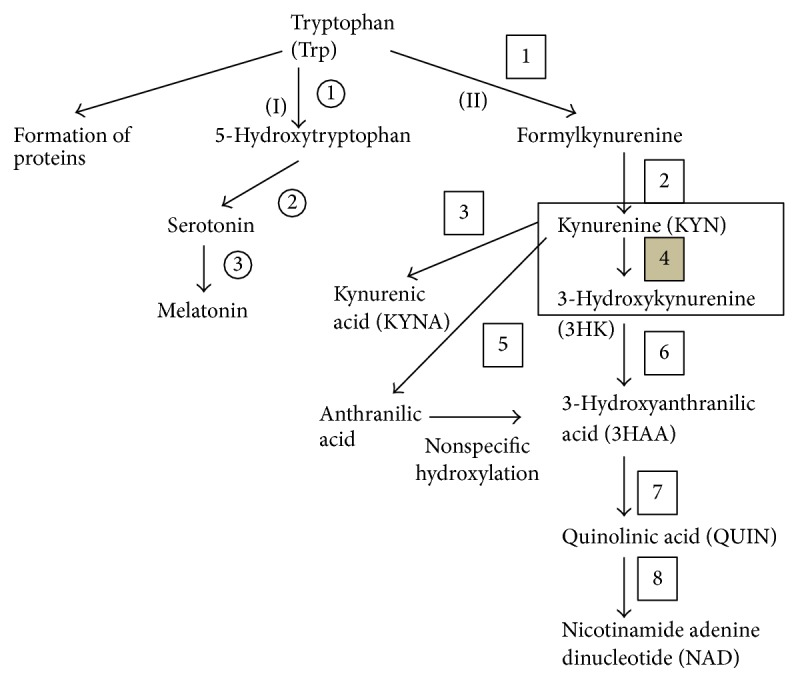
Tryptophan metabolism. (I) Serotonin pathway: 1: tryptophan hydroxylase, 2: L-aromatic amino acid decarboxylase, and 3: serotonin-N-acetyltransferase and hydroxyindole-O-methyltransferase; (II) kynurenine pathway: 1: tryptophan dioxygenase (TDO) and indoleamine 2,3-dioxygenase (IDO), 2: formamidase, 3: kynurenine aminotransferases (KATs), 4: kynurenine 3-monooxygenase (KMO), 5: kynureninase, 6: kynureninase, 7: 3-hydroxyanthranilic acid oxygenase, and 8: quinolinic acid phosphoribosyltransferase.

In the human brain, 95% of Trp is involved in the kynurenine pathway (KP) [11] (the rest 5% are involved in serotonin (5-hydroxytryptamine (5-HT)) pathway (SP) and the formation of proteins) (Figure 1). This enzymatic cascade is responsible for the synthesis of nicotinamide adenine dinucleotide (NAD) and NAD phosphate. Within the central nervous system, the infiltrating macrophages, activated microglia, and neurones are capable of the complete enzymatic cascade, whereas the astrocytes and oligodendrocytes lack kynurenine 3-monooxygenase (KMO) and indoleamine 2,3-dioxygenase (IDO) and are unable to produce the neurotoxic quinolinic acid (QUIN) [12]. The central metabolite of the KP is kynurenine (KYN), which is formed from Trp after two enzymatic steps (involving IDO/tryptophan 2,3-dioxygenase and formamidase). The kynurenic acid (KYNA) is synthesized after an irreversible transamination by kynurenine aminotransferases (KATs) from KYN. The other main branch of the cascade begins with formation of the neurotoxic 3-hydroxykynurenine (3-HK) by KMO. This enzyme, situated in the outer membrane of the mitochondria [13], is responsible for the neurotoxic branch, the concentration of KYNA depending indirectly on the activity of KMO. Kynureninase converts 3-HK to another neurotoxic metabolite, QUIN. These two well-known neurotoxic intermediates of the KP are free radical generators, and 3-HK plays a role in immune processes, while QUIN is an NMDA receptor agonist [9].

In recent years, increasing evidence has emerged from an association between PD and the disruption of the KP and the SP. Lower 5-HT, KYN, and KYNA concentrations were measured in the frontal cortex, putamen, and pars compacta of the substantia nigra of PD patients than in the controls [14], while the 3-HK concentration was increased in the PD group [14]. In PD, the serotoninergic axons are degenerated [15], and the 5-HT level in the CSF is decreased [16].

In two animal models of the PD, the KP is also affected [17–19]. Alterations in the KP in the peripheral organs have also been demonstrated in PD [20], these changes perhaps comprising part of a possible protective process. Moreover both* in vitro* [21] and* in vivo* [22, 23] experiments have demonstrated the neuroprotective impact of KYNA.

The KMO gene is located on the 1q42 chromosome. KMO is responsible for the synthesis of 3-HK, the neurotoxic metabolite of the cascade [14]. Pharmacological inhibition of KMO is known to induce the synthesis of KYNA, and a polymorphism of the KMO gene might similarly shunt the metabolism towards the neuroprotective compound.

We are not aware of previous studies of KMO single nucleotide polymorphisms (SNPs) in PD, though some KMO SNPs have been investigated in schizophrenia and bipolar disease [24–27]. Perturbation of the dopaminergic system and the KP has been demonstrated in schizophrenia, similarly as in PD, and this led us to examine the roles of the earlier investigated schizophrenia-associated SNPs in PD. In our study, we examined the distribution of the different SNPs (rs2050518, rs6661244, rs2275163, and rs1053230) of the KMO gene in the PD patient and control groups, as well as the potential impact of the SNPs on the age at onset.

If alterations in the KP may contribute to the pathogenesis of PD, influencing the cascade will perhaps be a promising therapeutic target [28–32]; therefore, that is not to be sneezed at.

## 2. Patients and Methods

### 2.1. PD Patients and Controls

All study participants gave their written informed consent. 105 unrelated PD patients (57 females and 48 males; average age: 66.42 ± 0.90 years; average age at onset of the disease: 58.81 ± 10.97 years) and 131 healthy volunteer controls (71 females and 60 males; average age: 65.21 ± 0.70 years) from Csongrád County, Hungary, were enrolled at the Department of Neurology, Faculty of Medicine, University of Szeged, and at the Department of Neurology and Cerebrovascular Diseases, Pándy Kálmán County Hospital, Gyula, Hungary. The patient and control groups did not differ in sex ratio (*P* = 0.989) or mean age (*P* = 0.069). For the age at onset investigations we divided our patient group into two (Group 1: the age at onset began <60, mean: 50.27 ± 7.734; Group 2: the age at onset began ≥60, mean: 67.70 ± 5.281). The general sociodemographic data are summarized in Table 1. The possible effects of four SNPs of the KMO gene (rs2050518, rs6661244, rs2275163, and rs1053230) were analysed. The rs1053230 SNP involves arginine (hydropathy index −4.5) and cysteine (hydropathy index 2.5) exchange. This change may affect the enzyme function, influencing substrate binding [15]. Carriers of the T allele of the rs2275163 SNP show a trend to an increased KMO mRNA level [26]. This may explain why the T allele carriers have a decreased KYNA concentration [25]. The two other SNPs are situated in the intronic segment of the gene. They may affect the regulatory protein binding site or may play a role in the regulation of the gene by microRNA. The study protocol was approved by the Medical Research Council Scientific and Research Ethics Committee (47066-3/2013/EKU (556/2013)) and was in full accordance with the Declaration of Helsinki.

### 2.2. Methods

#### 2.2.1. DNA Isolation

For genomic DNA isolation, peripheral blood was subjected to the standard desalting method of Miller et al. [33]. The purified genomic DNA was stored at −20°C until further use, at the biobank of the Department of Neurology, Faculty of Medicine, University of Szeged (biobank licence: Regional Human Biomedical Research Ethics Committee: 135/2008).

#### 2.2.2. Polymerase Chain Reaction with TaqMan Probes

Fluorescently labelled TaqMan probes were used for the allele discrimination. The designed primers and probes are summarized in Table 2.

For PCR amplification, the following parameters were used: 95°C for 3 min, followed by 49 cycles of 95°C for 10 s, and then 58°C for 50 s (except for rs2050518, where 59°C for 50 s was applied). A genotyping specific master mix was utilized from the PCR Biosystem (2x PCR Bio Genotyping mix Lo-ROX). The PCR experiments were performed with a BioRad CFX96 C1000 real-time thermal cycler machine, and the data analysis was carried out with BioRad software (BioRad CFX Manager version 1.6).

#### 2.2.3. Statistical Methods

For evaluation of the data, SPSS software version 20.0 was used. The chi-square test was utilized for comparison of the distributions of genotypes and alleles and the *t*-test to compare the averages in the two groups.

The observed genotype frequencies were in accordance with the Hardy-Weinberg equilibrium in both the PD and the control groups.

## 3. Results

We enrolled 105 PD patients and 131 healthy controls in our TaqMan probes allele discrimination KMO gene study. Three of the four investigated SNP are intronic variants and one involves a missense change in the genome.

### 3.1. rs2050518

This SNP, an A/T change, is localized in the intronic segment of the KMO gene. The genotype distribution in the PD patient group was 35 AA, 60 AT, and 10 TT, and that in the control group was 54 AA, 60 AT, and 17 TT. The allele frequencies in the two groups were very similar (Table 3). This SNP variant was not associated with the PD (genotype: *P* = 0.218, A allele: *P* = 0.408, and T allele: *P* = 0.214) and did not affect the age at disease onset (genotype: *P* = 0.977, A allele: *P* = 0.923, and T allele: *P* = 0.832).

### 3.2. rs6661244

This intronic variant of the KMO gene is a C/T change. The genotype distribution in the PD patient group was 37 CC, 58 CT, and 10 TT and in the control group was 54 CC, 61 CT, and 15 TT. The allele frequencies were 62.85% C allele and 37.14% T allele in the PD group and 64.88% C and 35.11% T in the controls (Table 3). This SNP variant was not associated with the PD (genotype: *P* = 0.481, C allele: *P* = 0.633, and T allele: *P* = 0.348) and did not affect the age at disease onset (genotype: *P* = 0.425, C allele: *P* = 0.446, and T allele *P* = 0.224).

### 3.3. rs2275163

This intronic variant of the KMO gene is another C/T change. The genotype distribution was 39 CC, 56 CT and 10 homozygote TT in the PD group and 55 CC, 61 heterozygote and 15 TT in the control one. The allele frequency results were 63.80% C allele in the PD group versus 65.26% C allele in the controls and 36.19% T allele in the PD group versus 34.73% T allele in the control group. This SNP variant was likewise not associated with PD (genotype: *P* = 0.581, C allele: *P* = 0.633, and T allele: *P* = 0.450) and did not affect the age at disease onset (genotype: *P* = 0.612, C allele: *P* = 0.446, and T allele: *P* = 0.669) (Table 3).

### 3.4. rs1053230

The last investigated SNP of the KMO gene involves a missense mutation (A/G) in exon 15. It results in an amino acid sequence shift from arginine to cysteine, which is localized in the outside part of the enzyme on the mitochondria membrane (https://www.predictprotein.org/), which is probably the site for substrate interaction, and this mutation might therefore directly influence the substrate binding characteristic of the protein. Our results indicated the lack of an association between rs1053230 and PD (genotype: *P* = 0.771, A allele: *P* = 0.710, and G allele: *P* = 0.485), and the mutation did not affect the age at disease onset either (genotype: *P* = 0.714, A allele: *P* = 0.960, and G allele: *P* = 0.442).

Thus none of the four investigated SNP polymorphisms were associated with PD or affected the age at disease onset (Table 3).

## 4. Discussion

This study related to the possibility of the predisposing roles of SNPs of the KMO gene in PD. To date there have been no genetic studies of the role of the KP in the pathogenesis of PD. None of the four investigated SNPs proved to be associated with PD or affected the age at onset. The impact of KMO polymorphism in the KP has been investigated only in schizophrenia and bipolar disease. There has been only one study which revealed an association between rs1053230 and the KYNA concentration in the CSF in schizophrenia [34]. One limitation of this work was the low sample size, and it is therefore suggested that the experiments should be repeated with an independent, large sample [34, 35].

In summary, this was the first investigation of the potential role of polymorphism of the KMO gene in PD. It emerged that the investigated SNPs presumably are not associated with PD and probably do not affect the age at disease onset either. The genetic link between the KP and PD is still missing. These investigated SNPs most likely do not influence the function of KMO and supposedly do not contain binding sites for regulatory proteins relevant in PD. In the future further studies with larger sample size are needed to investigate the effect of the different alleles, SNPs of the kynurenine pathway enzymes, and the epigenetic regulation of these enzymes in PD.

Although the number of samples involved in this study is not that large, it is the first study to assess the genetic background behind the biochemical alterations of the kynurenine pathway in PD, directing the attention to this previously unexamined field.

Alterations in this pathway are associated with a number of neurologic disorders, such as Alzheimer's disease, depression, and PD [10], and the ability to influence this enzyme pathway may have beneficial properties in these diseases.

## Figures and Tables

**Table 1:** Sociodemographic data of the PD patients and the controls.

Group	Males	Females	Mean	Median	Min	Max	Age of onset
PD (105)	48	57	66.42 ± 0.901	68	34	84	58.81 ± 10.970
HC (131)	60	71	65.21 ± 0.705	63	53	87	—

Min: minimum age in the group; Max: maximum age in the group.

**Table 2:** The summary of the designed primers and probes.

SNP	Primers	Probes
rs2050518	F: 5′-TCA TAT CAT ATC TCA CTG TGT GAA-3′	A allele: 5′-Fam-TCG TTC ATT CCA CTC TGA TAG TC-BHQ-1-3′
	R: 5′-CCA GGT TGT TCA GTG TAG T-3′	T allele: 5′-Hex-TCG TTC ATT CCT CTC TGA TAG TC-BHQ-1-3′

rs6661244	F: 5′-CAT GGC AAA TAC AAT GGC T-3′	C allele: 5′-Fam-AAT CTG AGG CCT ATG GTG ATG T-BHQ-1-3′
	R: 5′-ACA AAC ATA AAT CCT CTC TGG A-3′	T allele: 5′-Hex-AAT CTG AGG CTT ATG GTG ATG T-BHQ-1-3′

rs2275163	F: 5′-ACG ATG GAT CAT GCA GTA A-3′	C allele: 5′-Fam-TAG AGC AAA AGT CTA AGT GGA TAT TG-BHQ-1-3′
	R: 5′-CGT CAA GGG TGT TTT TCA G-3′	T allele: 5′-Hex-TAG AGC AAA AGT TTA AGT GGA TAT TG-BHQ-1-3′

rs1053230	F: 5′-TTT GCT ACC ACA AAA CCT TT-3′	A allele: 5′-Fam-CCT CTC AAG CAG AGG AAA GAT C-BHQ-1-3′
	R: 5′-TCA GCA GTA CCT ACC TAC TTA TA-3′	G allele: 5′-Hex-CTT CTC AAG CGG AGG AAA GAT C-BHQ-1-3′

F: forward primer; R: reverse primer.

**Table 3:** Genotype and allele distribution of the investigated SNPs.

		Genotype frequency			Allele frequency	
		AA	AT	TT	A	T
rs2050518	PD patients	35	60	10	130 (61.90%)	80 (38.09%)
	Controls	54	60	17	168 (64.12%)	94 (35.87%)

		Genotype frequency			Allele frequency	
		CC	CT	TT	C	T

rs6661244	PD patients	37	58	10	132 (62.85%)	78 (37.14%)
	Controls	54	62	15	170 (64.88%)	92 (35.11%)

		Genotype frequency			Allele frequency	
		CC	CT	TT	C	T

rs2275163	PD patients	39	56	10	134 (63.80%)	76 (36.19%)
	Controls	55	61	15	171 (65.26%)	91 (34.73%)

		Genotype frequency			Allele frequency	
		AA	AG	GG	A	G

rs1053230	PD patients	7	38	60	52 (24.76%)	158 (75.23%)
	Controls	6	47	78	59 (22.51%)	203 (77.48%)

## Abbreviations

3-HK: 3-Hydroxykynurenine
5-HT: 5-Hydroxytryptamine, serotonin
CSF: Cerebrospinal fluid
IDO: Indoleamine 2,3-dioxygenase
KAT: Kynurenine aminotransferase
KMO: Kynurenine 3-monooxygenase
KP: Kynurenine pathway
KYN: Kynurenine
KYNA: Kynurenic acid
NAD: Nicotinamide adenine dinucleotide
PD: Parkinson's disease
QUIN: Quinolinic acid
SNP: Single nucleotide polymorphism
SP: Serotonin pathway
Trp: Tryptophan.
